# Long-term follow up of patients with hematological malignancies treated with total body irradiation using intensity modulated radiation therapy

**DOI:** 10.3389/fonc.2022.1044539

**Published:** 2022-12-02

**Authors:** Colton Ladbury, Claire Hao, Dongyun Yang, Susanta Hui, Chunhui Han, An Liu, Amandeep Salhotra, Ryotaro Nakamura, Joseph Rosenthal, Anthony Stein, Jeffrey Wong, Savita Dandapani

**Affiliations:** ^1^ Department of Radiation Oncology, City of Hope National Medical Center, Duarte, CA, United States; ^2^ Division of Biostatistics, City of Hope National Medical Center, Duarte, CA, United States; ^3^ Department of Hematology and Hematopoietic Cell Transplantation, City of Hope National Medical Center, Duarte, CA, United States

**Keywords:** total body irradiation (TBI), IMRT (intensity modulated radiation therapy), HSCT = hematopoietic stem cell transplant, tomotherapy, VMAT (volumetric modulated arc therapy)

## Abstract

**Background:**

With the advent of modern radiation treatment technologies such as intensity modulated radiation therapy (IMRT), there has been increasing interest in its use for total body irradiation (TBI) conditioning regimens for hematopoietic cell transplantation (HCT) to achieve lower doses to critical organs such as the lungs and kidneys. Although this has been reported on in early studies, long-term safety and efficacy data is limited.

**Methods:**

We performed a single institution matched-pair retrospective analysis of patients treated with IMRT TBI and standard TBI between 2010 and 2020 to provide data on long-term outcomes. Patients with hematologic malignancies, who could not tolerate standing for traditional TBI or who received prior radiation received IMRT TBI. Patients were matched based on age, diagnosis, disease status, and year of transplant, and were matched 2:1 to the standard TBI and IMRT TBI cohorts. Patient and treatment characteristics, toxicity, graft-versus-host disease (GVHD), dosimetry, and outcomes were evaluated for each cohort.

**Results:**

A total of 13 patients met inclusion criteria for the IMRT cohort, leading to 26 patients in the standard TBI cohort. There was no significant difference in relevant clinical factors between the cohorts. Reasons for using IMRT over conventional TBI included being unable to stand (n=5), prior radiation (n=5), and pediatric patient requiring anesthesia (n=3). Among living patients, median follow-up for all patients was 5.1 years in the IMRT TBI cohort and 5.5 years in the standard TBI cohort. The 5-yr estimate of OS was 68% in the IMRT TBI cohort and 60% in the standard TBI cohort (p=0.706). The 5-yr estimate of RFS was 54% in the IMRT TBI cohort and 60% in the standard TBI cohort (p=0.529). There was no clinically significant pneumonitis, nephritis, hypothyroidism, or cataracts reported in the IMRT TBI cohort. 41.7% of patients in the IMRT TBI cohort and 79.2% of patients in the standard TBI cohort experienced Grade II-IV acute GVHD (p=0.023).

**Conclusions:**

IMRT TBI appears to lead to favorable long-term outcome and dosimetry, and therefore potentially improved long-term toxicity profile compared to conventional TBI. IMRT TBI warrants further investigation as part of larger prospective trials.

## Introduction

Since the 1970s, total body irradiation (TBI) has played a central role in conditioning regimens prior to hematopoietic stem cell transplant (HSCT) (1, 2). The goal of TBI is to ablate any remaining malignant cells and/or to reduce risk of transplant rejection by modulating the immune system. Historically, TBI has been administered using 2-dimensional planning, which limits sparing of normal organs, and leads to significant possible adverse toxicity. Indeed, typically attempts are only made to spare the lung by using lung blocks (3). However, even that methodology is suboptimal. In an analysis of a Children’s Oncology Group (COG) trial, lung dose <8 Gy was associated with improved overall survival, but can be difficult to achieve with conventional TBI (4). Furthermore, the analysis found that lung shielding during TBI is not standardized, with lung doses ranging from 50% to the full TBI dose, justifying the need for more modern radiation techniques to reduce dose to organs at risk (OARs).

One such approach has been to use a more modern 3-dimensional radiation technique in intensity modulated radiation therapy (IMRT) to deliver TBI, which has the capability of delivering a more sculpted and conformal dose distribution. The utility of such an approach has previously been described, with tomotherapy (Tomo IMRT) reducing the mean lung dose from 8-9 Gy to 5-6 Gy, and reduced dose correlating with significantly less pulmonary complications in patients treated with total marrow and lymphoid radiation (TMLI) (5, 6). Multiple trials have reported on using IMRT TBI, using either a Tomo IMRT (7) (8) or volumetric modulated arc therapy (VMAT) technique (9, 10). However, given the recent publication of IMRT TBI studies, there is a paucity of long-term safety and efficacy data.

Recent retrospective studies have offered some insight into the long-term outcomes of IMRT TBI. One Russian study of 220 pediatric patients who received TBI- and chemo-conditioning for allogeneic HSCT with TCRαβ/CD19 depletion reported an overall survival (OS) of 63% and event-free survival (EFS) of 58% at a median follow-up of 2.8 years (11). Another retrospective of 44 pediatric and adult patients at UT Southwestern who received low- and standard-dose VMAT-TBI found that median time to relapse was 11.3 months at a follow up of 2.2 years, and reported that acute and chronic GVHD occurred in 59% and 39% of patients, respectively (12). Most recently, researchers at Stanford reported on a group of 38 pediatric and adolescent/young adult (AYA) patients who received VMAT-TBI at both myeloablative and non-myeloablative doses. At a median follow-up of 8.7 months, they reported an OS of 89.5% and relapse-free survival (RFS) of 94.7% for all patients (13). While these studies emphasize the safety and efficacy of IMRT TBI, their median follow-up periods do not exceed three years, limiting our understanding of the long-term effects of IMRT TBI.

Since 2010, our institution has been performing Tomo IMRT TBI off-protocol for patients who cannot tolerate standing for treatment or who have had prior radiation. Therefore, we performed a retrospective matched-pair analysis of the treatment and outcomes of these patients compared to patients who received IMRT TBI. Herein, we report on their long-term follow-up, including oncologic outcomes and associated adverse effects.

## Materials and methods

### Study design

The present study is a single-center matched-pair retrospective analysis of patients treated with HSCT. The conditioning regimen in all patients included TBI. Patients were matched with a 2:1 ratio between having TBI delivered by standard techniques or IMRT. Endpoints included relapse-free survival (RFS), overall survival (OS), the incidence of late toxicities based on the National Cancer Institute Common Terminology Criteria for Adverse Events (CTCAE) version 4.0, the incidence of acute or chronic graft-versus-host disease (aGVHD or cGVHD, respectively), and GVHD-free relapse-free survival (GRFS). GVHD was scored by the Glucksberg system (14). This study was registered with and approved by the City of Hope National Medical Center Institutional Review Board.

### Patients

Patients were treated between 2010 and 2020. Only patients who could not tolerate standing for TBI or who had received prior radiation were eligible for the IMRT TBI cohort. Patients with diagnoses of acute lymphocytic leukemia (ALL), acute myeloid leukemia (AML), biphenotypic acute leukemia (BAL), and NHL) were included. For patients with acute myeloid leukemia (AML) or acute lymphoblastic leukemia (ALL), standard TBI matches were made based on age (<20, 20-39, 40+), diagnosis (AML or ALL), complete response (CR) status (CR1, CR2, CR3+), and HCT period (2010-2016, 2017-2020). Due to smaller patient numbers, for patients with biphenotypic acute leukemia or NHL, standard TBI matches were made based on age (<40, 40+) and CR status (CR1 or CR2, CR3+).

### IMRT TBI treatment regimen

**Figure 1:**
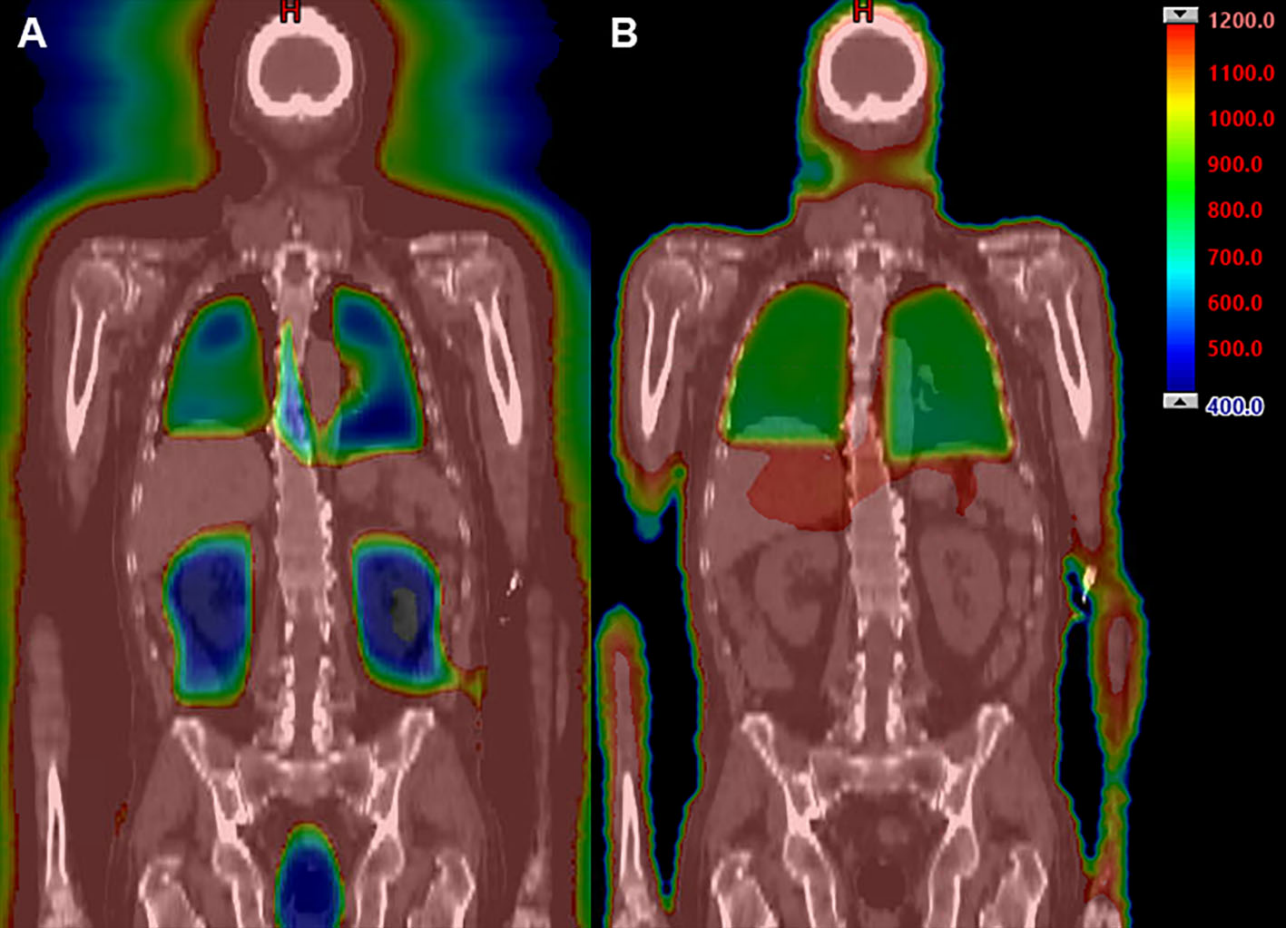
Example dose distribution (range 400 cGy [blue] to 1200 cGy [red]) using IMRT TBI **(A)** compared to standard TBI techniques **(B)**.

All patients underwent scanning with a large-bore computed tomography (CT) simulator with 60-cm field of view (Phillips Medical System, Eindhoven, Netherlands) for treatment planning purposes. Scans were obtained during shallow breathing, inspiration, and expiration to account for organ motion due to respiration. Patients were immobilized using a full-body Vac-lok bag (Civco Medical Systems, Kalona, IA) and a thermoplastic mask on the head and neck region. Treatment planning CT images were obtained with a slice thickness of 5 mm. The clinical target volume (CTV) was defined as the entire body minus the lungs. The prescription target volume (PTV) was equal to the CTV without additional expansion. The minimum PTV dose was to be 80% of prescription dose, with maximum dose ≤130%. The prescribed radiation dose varied based on regimen and indication, ranging from 1200-1350 cGy in 120-165 cGy fractions, administered 2-3 times daily with the interval between fractions being greater than 6 hours. Dose constraints included a mean lung dose of ≤800 cGy and mean kidney dose ≤1200 cGy. The brain, lens, and oral cavity were spared in one, two, and three patients, respectively based on prior radiation exposure. An example IMRT TBI plan is shown in **Figure 1A** compared to a standard IMRT TBI plan is shown in **Figure 1B**. All patients were treated with a helical TomoTherapy unit (Accuray, Inc, Sunnyvale, CA), and the lower extremities were treated with a conventional linear accelerator through standard anteroposterior posteroanterior fields with matching fields given limitation of field size on the linear accelerator. TBI was delivered with image-guided radiation therapy, and mega-voltage CT was used with each fraction to align patients.

### Systemic therapies

The systemic conditioning regimen was administered at the discretion of the treating hematologist. Patients were treated with some combination of cyclophosphamide (CTX), etoposide (VP-16), fludarabine (FLU), and melphalan (MEL). Standard GVHD prophylaxis consisted of some combination of cyclosporine, tacrolimus, sirolimus, methotrexate (MTX), post-HSCT CTX, and mycophenolate mofetil. The institutional supportive care regimen was used to manage nausea, vomiting, mucositis and infection risks.

### IMRT TBI dosimetry

IMRT treatment plans that were generated using the TomoTherapy system were imported into the Eclipse planning system for analysis. Dosimetric parameters from the PTV and relevant OARs were retrospectively extracted from each patient’s treatment plan using the Eclipse (Varian Medical Systems, Palo Alto, CA) planning system. Examined parameters included treatment time, mean doses, max doses, and volume of structure receiving a given dose. All mean and median doses were normalized to the prescription dose for uniformity.

### Statistical considerations

Patient and treatment characteristics, toxicity, and dosimetry were tabulated and evaluated using descriptive statistics. Due to low incidences of late toxicity and small patient numbers, rates of toxicities were not compared quantitatively between cohorts. Comparisons between the IMRT TBI and the standard TBI groups were made using Fisher’s exact test for categorical variables and Wilcoxon two-group test for continuous variables. Follow-up data were collected through August 2022. Survival outcomes included overall survival (OS) and recurrence-free survival (RFS) and were calculated from the date of transplant. For OS, data for patients who were still alive were censored at the date of last follow-up. For RFS, the events included death or disease recurrence, whichever came first. For GRFS, events included death, disease recurrence, or development of grade III-IV aGVHD or extensive cGVHD. Data for patients who did not experience disease recurrence and were still alive were censored at the date of last follow-up. Survival rates were estimated using the Kaplan-Meier method. Incidence of adverse events and GVHD was tabulated by classification and grade. Comparisons of GVHD incidence were performed using Gray’s k-sample test for equality of cumulative incidence functions. All statistical analyses were performed using Python 3.10 (PSF, Wilmington, DE).

## Results

### Patient characteristics

Patient characteristics are presented in **Table 1**. Reasons for using IMRT over conventional TBI included being unable to stand (n=5), prior radiation (n=5), and pediatric patient requiring anesthesia (n=3). A total of 13 patients were identified as meeting inclusion criteria for the IMRT TBI cohort, with 26 matched standard TBI patients. The median age of the patients was 31 years (6-52 years) in the IMRT TBI cohort and 34 years (11-56) in the standard TBI cohort (p=0.61). The most common diagnoses were AML (53.8%) and ALL (38.5%). There was no difference in relevant clinical factors including sex (0.73), Karnofsky performance score (p=0.26), disease status (p=0.72), transplant period (p=0.52), conditioning regimen (p=0.75), or GVHD prophylaxis (p=0.23).

**Table 1:** Patient and treatment characteristics.

	IMRT TBI (N = 13)	Standard TBI (N = 26)	Total (N = 39)	p
Age at transplant, years				0.61
Median	31	34	33	
Interquartile range	11, 46	21, 45	20, 46	
Range	(6-52)	(11-56)	(6-56)	
Sex				0.73
Male	8 (61.5%)	13 (50%)	21 (53.8%)	
Female	5 (38.5%)	13 (50%)	18 (46.2%)	
Karnofsky performance status				0.26
80-100	10 (76.9%)	24 (92.3%)	34 (87.2%)	
≤70	2 (15.4%)	2 (7.7%)	4 (10.3%)	
Unknown	1 (7.7%)	0 (0%)	1 (2.6%)	
HCT comorbidity index				0.52
0	4 (30.8%)	10 (38.5%)	14 (35.9%)	
1-2	2 (15.4%)	7 (26.9%)	9 (23.1%)	
≥3	7 (53.8%)	9 (34.6%)	16 (41%)	
Diagnosis				1.00
ALL	5 (38.5%)	10 (38.5%)	15 (38.5%)	
AML	5 (53.8%)	10 (53.8%)	15 (53.8%)	
Non-Hodgkin’s Lymphoma	1 (7.7%)	2 (7.7%)	3 (7.7%)	
Biphenotypic Leukemia	2 (15.4%)	4 (15.4%)	6 (15.4%)	
Disease status				0.72
1st CR	7 (53.8%)	17 (65.4%)	24 (61.5%)	
2nd CR	4 (30.8%)	5 (19.2%)	9 (23.1%)	
>=3rd CR	2 (15.4%)	4 (15.4%)	6 (15.4%)	
Donor type				0.84
Matched related	3 (23.1%)	10 (38.5%)	13 (33.3%)	
Matched unrelated	6 (46.2%)	7 (26.9%)	13 (33.3%)	
Mismatched unrelated	2 (15.4%)	4 (15.4%)	6 (15.4%)	
Cord	1 (7.7%)	3 (11.5%)	4 (10.3%)	
Auto	1 (7.7%)	2 (7.7%)	3 (7.7%)	
Transplant period				0.52
2010-2016	6 (46.2%)	15 (57.7%)	21 (53.8%)	
2017-2020	7 (53.8%)	11 (42.3%)	18 (46.2%)	
Conditioning regimen				0.75
fludarabine/cyclophosphamide	1 (7.7%)	3 (11.5%)	4 (10.3%)	
fludarabine	0 (0%)	4 (15.4%)	4 (10.3%)	
cyclophosphamide	7 (53.8%)	10 (38.5%)	17 (43.6%)	
etoposide	4 (30.8%)	7 (26.9%)	11 (28.2%)	
etoposide/cyclophosphamide	1 (7.7%)	2 (7.7%)	3 (7.7%)	
Total radiation dose				0.18
1200	1 (7.7%)	6 (23.1%)	7 (17.9%)	
1320	11 (84.6%)	20 (76.9%)	31 (79.5%)	
1350	1 (7.7%)	0 (0%)	1 (2.6%)	
Stem cell source				0.87
Peripheral Stem Cells	8 (61.5%)	18 (69.2%)	26 (66.7%)	
Bone Marrow	4 (30.8%)	5 (19.2%)	9 (23.1%)	
Cord Blood	1 (7.7%)	3 (11.5%)	4 (10.3%)	
GVHD prophylaxis†				0.23
cyclophosphamide/tacrolimus/cellcept	0 (0%)	4 (16.7%)	4 (11.1%)	
cyclosporine/cellcept	1 (8.3%)	2 (8.3%)	3 (8.3%)	
cyclosporine/methotrexate	0 (0%)	1 (4.2%)	1 (2.8%)	
tacrolimus/cellcept	1 (8.3%)	0 (0%)	1 (2.8%)	
tacrolimus/methotrexate	4 (33.3%)	4 (16.7%)	8 (22.2%)	
tacrolimus/sirolimus	5 (41.7%)	13 (54.2%)	18 (50%)	
tacrolimus/sirolimus/methotrexate	1 (8.3%)	0 (0%)	1 (2.8%)	
Reason receiving IMRT				-
Originally been planned for TMLI	1 (7.7%)	-	-	
Pediatric	2 (15.4%)	-	-	
Prior radiation	4 (30.8%)	-	-	
Prior radiation & Pediatric	1 (7.7%)	-	-	
Unable to stand	5 (38.5%)	-	-	

† Applicable to alloHCT only.

ALL, acute lymphoblastic leukemia; AML, acute myeloid leukemia; CR, complete remission; TMLI, total marrow and lymphoid radiation; IMRT, intensity modulated radiation therapy; GVHD, graft-versus-host disease.

### Clinical outcomes

**Figure 2:**
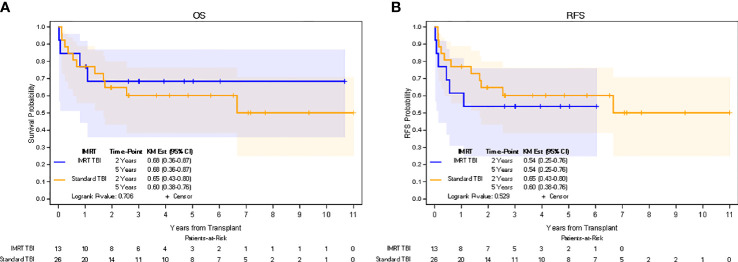
Kaplan Meier estimates of overall survival **(A)** and recurrence-free survival **(B)**.

In the IMRT TBI cohort, median follow-up for all patients was 4.0 years (0.1-10.7 years), while median follow-up for living patients was 5.1 years (2.1-10.7 years). In the standard TBI cohort, median follow-up for all patients was 2.7 years (0.1-11.0 years), while median follow-up for living patients was 5.5 years (0.2-11.0 years). The 5-yr estimate of OS was 68% (95% CI: 36-87%) in the IMRT TBI cohort and 60% (95% CI: 38%-76%) in the standard TBI cohort (p=0.706). The 5-yr estimate of RFS was 54% (95% CI: 25%-76%) in the IMRT TBI cohort and 60% (95% CI: 38%-76%) in the standard TBI cohort (p=0.529). The 5-yr estimate of GRFS was 31% (95% CI: 10%-45%) in the IMRT TBI cohort and 25% (95% CI: 8%-39%) in the standard TBI cohort (p=0.652). All relapses in the IMRT TBI cohort were limited to the bone marrow. Kaplan-Meier estimates for OS and RFS are shown in **Figure 2**. Of the four patients who died in the IMRT arm, only one died of relapse. Other causes of death included neutropenic fever/sepsis, epilepsy, and respiratory failure, of which the first two occurred within a month of transplantation.

### Engraftment, adverse events, and GVHD

All patients experienced expected cytopenias that resolved, and all patients successfully engrafted a median of 16 days following the transplant. On long-term follow-up, no patients in the IMRT TBI cohort reported clinically significant pneumonitis, nephritis, or cataracts. In the standard TBI cohort, three (11.5%) patients developed cataracts, and five (19.2%) experienced pneumonitis. A total of three (23.1%) patients in the IMRT TBI cohort had transplant course complicated by infection, compared to six (23.1%) in the standard TBI cohort. No patients in either cohort experienced subsequent malignant neoplasms nor significant transplant-related morbidity other than the aforementioned toxicities. Five (41.7%) patients in the IMRT TBI cohort and 19 (79.2%) patients in the standard TBI cohort experienced Grade II-IV aGVHD (p=0.023). Four (33.3%) patients in the IMRT TBI cohort and eight (33.3%) patients in the standard TBI cohort experienced Grade III-IV aGVHD (p=0.614). Six (50.0%) patients in the IMRT TBI cohort and 13 (56.5%) patients in the standard TBI cohort experienced extensive cGVHD (p=0.801). No patients experienced limited cGVHD.

### Dosimetry

A summary of dosimetric parameters for the IMRT TBI plans is shown in **Table 2**. Median treatment time was 23.0 minutes (14.2-30.1 minutes). The median PTV V100 was 85.3% (77.1-91.4%), with mean dose to the PTV being 102.2% of the prescription dose (97.8-131.7%). Mean hotspot was 120.5% of prescription dose (111.3-141.6%). Mean lung dose was 47.0% of prescription dose (35.7-62.6%) or 622.6 cGy (471.8-783.5 cGy) and mean dose to kidneys was 44.1% of prescription dose (27.5-105.3%) or 565.9 cGy (363.0-1263.9 cGy). There was significant variation in brain, lens, and oral cavity dose, as these were selectively spared in some patients and treated to full dose in other patients.

**Table 2:** IMRT TBI Dosimetric parameters.

Characteristic	% Rx Median (range)	cGy Median (range)
Treatment Time (minutes)	23.0 (14.2-30.1)	
PTV V100 (%)	85.3 (77.1-91.4)	
PTV Dmean	102.2 (97.8-131.7)	1343.6 (1208.5-1738.1)
PTV Dmax	120.5 (111.3-141.6)	1582.1 (1440.3-1869.7)
Lung Dmean	47.0 (35.7-62.6)	622.6 (471.8-783.5)
Lung V8 (%)	19.2 (8.3-43.2)	
Kidney Dmean	44.1 (27.5-105.3)	565.9 (363-1263.9)
Brain Dmean	103.5 (2.5-105.5)	1356.8 (33.3-1423.8)
Brain Dmax	106.6 (8.6-111.0)	1406.9 (113.2-1465.2)
Lens Dmean	103.7 (3.6-106.2)	1368.8 (47.5-1431.9)
Lens Dmax	105.0 (4.0-110.2)	1383.9 (52.2-1454.7)
Spinal Cord Dmax	109.0 (106.5-114.5)	1438.3 (1296.9-1511.9)
Esophagus Dmax	113.5 (105.7-126.4)	1487.6 (1276.5-1669)
Oral Cavity Dmean	103.2 (35.4-105.5)	1350.5 (467.8-1424.1)
Oral Cavity Dmax	106.2 (80.6-109.6)	1400.5 (1063.4-1449.5)

Rx, prescription dose; cGy, centigray; VX, volume receiving at least X Gy; Dmean, mean dose; Dmax, max dose.

## Discussion

Advances in radiation technology have led to implementation of more advanced radiation techniques such as IMRT into the transplant conditioning regimen. Several studied have reported on the procedure for performing IMRT TBI, including initial clinical outcomes (11–13). However, among these studies, the highest median follow-up was 2.8 years, with a maximum follow up of 6.4 years (11). Therefore, there is a need for evaluation of long-term clinical outcomes and toxicities. To our knowledge, our study offers the longest follow-up of patients treated with IMRT TBI, with a median follow-up of 5.1 years for living patients and a maximum follow-up of 10.7 years. In our study, we observe favorable long-term outcomes and rates of toxicity in our IMRT TBI cohort, which should support the continued investigation of IMRT TBI as a conditioning regimen for patients undergoing hematopoietic cell transplantation.

The improved ability of IMRT TBI to spare critical organs may help mitigate toxicities. Although there were variations in prescription dose, all patients in our cohort achieved the recommended mean lung doses of <800 cGy, which has been shown to be difficult to achieve with conventional blocks (4, 15). The value of this is illustrated by the lack of pneumonitis in our IMRT TBI cohort, while there were several cases in the standard TBI cohort. Similarly, almost all patients achieved a mean kidney dose of less than <12 Gy, with no nephrotoxicity events, though this was not unexpected, with low rates at baseline and no nephrotoxicity in the standard cohort either. Overall, our dosimetry is similar to results reported by Marquez et al., who achieved mean lung dose of 7.3 Gy and mean kidney dose of 71.4% of prescription dose, and also experienced no pneumonitis or nephritis (13). In fact, our mean doses of 6.2 Gy and 5.7 Gy respectively reflect potential to further limit exposure of critical organs, which may improve transplant-related morbidity and mortality (16). Our organ sparing still yielded high PTV coverage, with a median of 85% achieving prescription dose, and led to favorable oncologic outcomes.

Importantly, the 5-yr survival outcomes in our study does not appear to be negatively impacted by organ sparing. Indeed, we observed no recurrences nor deaths beyond two years and survival was not significantly different from the matched standard TBI cohort. Although there were two cases of transplant-related mortality within a month of transplant in the IMRT cohort, these deaths were attributed to neutropenic fever/sepsis and seizures, which are almost certainly unrelated to the TBI technique. Conditioning regimen is a critical contributor to risk of early relapse following transplant, so deintensifications such as IMRT TBI should be assessed critically (17). In total, there were three relapses, all of which were limited to the bone marrow and got the full radiation dose. Therefore, these relapses are almost certainly unrelated to TBI technique. Further, although heterogeneity across published and our own IMRT TBI cohorts limits cross study comparisons of survival, our 2-year outcomes are comparable to other reported series, where 2-yr OS varied from 63-79% and EFS ranged from 58-71% (11, 12).

Although other organs, such as the lens and oral cavity were only spared in select cases, the minimum doses to those structures were 3.6% and 35.4% or prescription dose, respectively. Such doses would not be feasible with traditional TBI. With limited patient numbers, we cannot be certain of the impact this sparing might have on long-term toxicity such as cataract formation (notably no patients in our IMRT cohort reported cataract development over the course of follow-up), though analyses of dose response for radiation cataractogenesis support this hypothesis (18). Although there were late toxicities observed in the standard TBI cohort, due to retrospective nature, low event rates, and small sample sizes, it is not possible to make conclusions on whether IMRT permits lower toxicity rates, though with organ sparing it is plausible and has been seen in related work (6).

Although currently not part of standard IMRT-TBI protocols, the ability to selectively target radiation dose and spare organs such as the lenses or oral cavity as part of the conditioning regimen allows for potential dose escalation of higher-risk areas while still sparing critical organs. This framework has provided the basis for similar work in the form of total marrow and lymphoid irradiation (TMLI), which had initially been used exclusively for high-risk patient populations who otherwise would not be transplant candidates (16, 19–21), but is now proceeding forward with trials in standard risk disease. Analysis of patients undergoing TMLI suggests organ sparing can lead to decreased toxicity in the form of lower rates of pneumonitis, nephrotoxicity, hypothyroidism, and cataract formation (6). Future studies involving IMRT TBI and TMLI will continue to seek to shape dose to optimize achieving disease control while limiting toxicities.

The incidence of GVHD, particularly in the standard TBI cohort, is relatively high compared to other more modern reports, though the etiology of this difference is unclear, given standard GVHD prophylaxis was administered and the vast majority of patients had matched donors (22, 23). Further, our institutional GVHD prophylaxis protocols are consistent with current recommended treatment standards. Overall, the rates of GVHD in our IMRT TBI cohort were similar to rates published by Zhang-Velten et al. (12) who also used IMRT TBI, as well as other non-IMRT TBI based regimens (24–27). There was also no difference in Grade III-IV aGVHD nor cGVHD in our standard TBI cohort compared to the IMRT TBI cohort. Interestingly, the IMRT TBI cohort did have significantly less grade II-IV aGVHD than the standard TBI cohort. The reason for this difference is not clear, however, there is an association between intensity of conditioning regimen and incidence of grade II-IV aGVHD (28). While the difference could be driven by differences in GVHD prophylaxis, patient population (though there were no significant differences in patient characteristics) or chance, it is possible that increased dose homogeneity and decreased hotspots in organs, such as the gastrointestinal tract, could be a cause (29). This would be an artifact caused by IMRT optimizations, given only lungs and kidney were contoured and intentionally spared. Interestingly, post-transplant cyclophosphamide may decrease aGVHD, though only patients in the standard TBI cohort received it (30, 31). Another possibility is contrast in dose rate between the techniques and associated differences in normal tissue damage from radiation (32). However, helical tomotherapy is associated with a dose rate much higher than standard TBI (33, 34), which one would expect to lead to greater toxicity, although this has not occurred in initial studies (6, 35, 36). Clearly, a better understanding of the relationship between radiation technique, dose rate, and transplant related morbidity will be necessary. Preclinical studies suggest aGVHD associated with radiation exposure level to associated organs, which can be attenuated using TMI (37–39). Future larger studies of IMRT TBI will optimally shed further light on whether technique might influence aGVHD.

Our study is limited by several features including small sample size and retrospective nature. Our small sample size also had significant heterogeneity, including multiple different hematologic malignancies and disease statuses at the time of transplant. This limits comparison of oncologic outcomes to other cohorts. We did seek to ameliorate this limitation by performing a matched pair analysis with patients receiving conventional TBI, which did not show any signal of any detriment to oncologic outcomes. The limitation also applies to assessment of late toxicity. Due to small patient numbers and the relative rarity of late toxicity events even in the general TBI population, it is difficult to draw definitive conclusions. This limitation is amplified by the retrospective nature of our study, since toxicities were not prospectively collected, and therefore had to be identified *via* chart review, which might miss some toxicities and generally precludes grading of toxicities. This also limits characterization of other non-malignant late effects and health-related quality of life. Nonetheless, the absence of significant late toxicities in our IMRT TBI cohort is promising. Despite these limitations, our study adds to the literature on IMRT TBI by providing some insight into long-term follow-up.

## Conclusions

IMRT TBI appears to lead to favorable long-term outcomes, with a potential for improved long-term toxicity profile compared to conventional TBI associated with better organ sparing. Based on the results of our initial work, we have implemented a multi-institution pilot study (NCT04281199) of TBI using tomotherapy or VMAT-based IMRT in order to achieve new lung dose guidelines (4). We have also initiated a protocol for the treatment of scleroderma following positive results from the SCOT trial (40). IMRT TBI warrants further investigation as part of larger prospective trials.

## Data availability statement

The raw data supporting the conclusions of this article will be made available by the authors, without undue reservation.

## Ethics statement

The studies involving human participants were reviewed and approved by City of Hope National Medical Center Institutional Review Board. Written informed consent from the participants’ legal guardian/next of kin was not required to participate in this study in accordance with the national legislation and the institutional requirements.

## Author contributions

CL and CIH performed data collection, data analysis, and manuscript preparation. DY participated in the design of this study and performed data collection and data analysis. SD participated in the design of this study, creation of methods for this study, and data analysis, and performed critical review of the manuscript. All authors contributed to the article and approved the submitted version.

## Conflict of interest

The authors declare that the research was conducted in the absence of any commercial or financial relationships that could be construed as a potential conflict of interest.

## Publisher’s note

All claims expressed in this article are solely those of the authors and do not necessarily represent those of their affiliated organizations, or those of the publisher, the editors and the reviewers. Any product that may be evaluated in this article, or claim that may be made by its manufacturer, is not guaranteed or endorsed by the publisher.

## References

[1] Wong JYC Filippi AR Dabaja BS Yahalom J Specht L . Total body irradiation: Guidelines from the international lymphoma radiation oncology group (ILROG). Int J Radiat Oncol Biol Phys (2018) 101(3):521–9. doi: 10.1016/j.ijrobp.2018.04.071 29893272

[2] Paix A Antoni D Waissi W Ledoux MP Bilger K Fornecker L . Total body irradiation in allogeneic bone marrow transplantation conditioning regimens: A review. Crit Rev Oncol Hematol (2018) 123:138–48. doi: 10.1016/j.critrevonc.2018.01.011 29482775

[3] Tas B Durmus IF Okumus A Uzel OE Gokce M Goksoy HS . Total-body irradiation using linac-based volumetric modulated arc therapy: Its clinical accuracy, feasibility and reliability. Radiother Oncol (2018) 129(3):527–3. doi: 10.1063/1.4976463 30172456

[4] Esiashvili N Lu X Hunger S Merchant TE Brown PA Wall DA . Association of higher lung dose received during total body irradiation for allogeneic hematopoetic stem cell transplantation in children with acute lymphoblastic leukemia with inferior progression-free and overall survival: A report from the children's oncology group. J Clin Oncol (2015) 33:15_suppl, 10030. doi: 10.1200/jco.2015.33.15_suppl.10030 PMC6548591 30807822

[5] Zhuang AH Liu A Schultheiss TE Wong JY . Dosimetric study and verification of total body irradiation using helical tomotherapy and its comparison to extended SSD technique. Med Dosim (2010) 35(4):243–9. doi: 10.1016/j.meddos.2009.07.001 19944588

[6] Shinde A Yang D Frankel P Liu A Han C Del Vecchio B . Radiation-related toxicities using organ sparing total marrow irradiation transplant conditioning regimens. Int J Radiat OncolBiolPhys (2019) 105(5):1025–33. doi: 10.1016/j.ijrobp.2019.08.010 31421151

[7] Gruen A Ebell W Wlodarczyk W Neumann O Kuehl JS Stromberger C . Total body irradiation (TBI) using helical tomotherapy in children and young adults undergoing stem cell transplantation. Radiat Oncol (2013) 8:92. doi: 10.1186/1748-717X-8-92 23587349 PMC3653702

[8] Sarradin V Simon L Huynh A Gilhodes J Filleron T Izar F . Total body irradiation using helical tomotherapy. Cancer Radiother (2018) 22(1):17–24. doi: 10.1016/j.canrad.2017.06.014 29395854

[9] Aydogan B Mundt AJ Roeske JC . Linac-based intensity modulated total marrow irradiation (IM-TMI). Technol Cancer Res Treat (2006) 5(5):513–19. doi: 10.1177/153303460600500508 16981794

[10] Aydogan B Yeginer M Kavak GO Fan J Radosevich JA Gwe-Ya K . Total marrow irradiation with RapidArc volumetric arc therapy. Int J Radiat Oncol Biol Phys (2011) 81(2):592–9. doi: 10.1016/j.ijrobp.2010.11.035 21345619

[11] Kobyzeva D Shelikhova L Loginova A Kanestri F Tovmasyan D Maschan M . Optimized conformal total body irradiation among recipients of TCRαβ/CD19-depleted grafts in pediatric patients with hematologic malignancies: Single-center experience. Front Oncol (2021) 11:785916. doi: 10.3389/fonc.2021.785916 34976825 PMC8716385

[12] Zhang-Velten ER Parsons D Lee P Chambers E Abdulrahman R Desai NB . Volumetric modulated arc therapy enabled total body irradiation (VMAT-TBI): Six-year clinical experience and treatment outcomes. Transplant Cell Ther (2022) 28(2):113.e1–.e8. doi: 10.1016/j.jtct.2021.10.020 34775145

[13] Marquez C Hui C Simiele E Blomain E Oh J Bertaina A . Volumetric modulated arc therapy total body irradiation in pediatric and adolescent/young adult patients undergoing stem cell transplantation: Early outcomes and toxicities. Pediatr Blood Cancer (2022) 69(6):e29689. doi: 10.1002/pbc.29689 35373904

[14] Glucksberg H Storb R Fefer A Buckner CD Neiman PE Clift RA . Clinical manifestations of graft-versus-host disease in human recipients of marrow from HL-a-matched sibling donors. Transplantation (1974) 18(4):295–304. doi: 10.1097/00007890-197410000-00001 4153799

[15] Luk SM Wallner K Glenn MC Ermoian R Phillips MH Tseng YD . Effect of total body irradiation lung block parameters on lung doses using three-dimensional dosimetry. J Appl Clin Med Phys (2022) 23(4):e13513. doi: 10.1002/acm2.13513 34985180 PMC8992940

[16] Wong JYC Liu A Schultheiss T Popplewell L Stein A Rosenthal J . Targeted total marrow irradiation using three-dimensional image-guided tomographic intensity-modulated radiation therapy: An alternative to standard total body irradiation. Biol Blood Marrow Transplant (2006) 12(3):306–15. doi: 10.1016/j.bbmt.2005.10.026 16503500

[17] Tauro S Craddock C Peggs K Begum G Mahendra P Cook G . Allogeneic stem-cell transplantation using a reduced-intensity conditioning regimen has the capacity to produce durable remissions and long-term disease-free survival in patients with high-risk acute myeloid leukemia and myelodysplasia. J Clin Oncol (2005) 23(36):9387–93. doi: 10.1200/JCO.2005.02.0057 16314618

[18] Hall MD Schultheiss TE Smith DD Nguyen KH Wong JY . Dose response for radiation cataractogenesis: A meta-regression of hematopoietic stem cell transplantation regimens. Int J Radiat Oncol Biol Phys (2015) 91(1):22–9. doi: 10.1016/j.ijrobp.2014.07.049 25227496

[19] Somlo G Spielberger R Frankel P Karanes C Krishnan A Parker P . Total marrow irradiation: A new ablative regimen as part of tandem autologous stem cell transplantation for patients with multiple myeloma. Clin Cancer Res (2011) 17(1):174–82. doi: 10.1158/1078-0432.CCR-10-1912 PMC3717559 21047977

[20] Jensen LG Stiller T Wong JYC Palmer J Stein A Rosenthal J . Total marrow lymphoid Irradiation/Fludarabine/ melphalan conditioning for allogeneic hematopoietic cell transplantation. Biol Blood Marrow Transplant (2018) 24(2):301–7. doi: 10.1016/j.bbmt.2017.09.019 29032268

[21] Wong JYC Filippi AR Scorsetti M Hui S Muren LP Mancosu P . Total marrow and total lymphoid irradiation in bone marrow transplantation for acute leukaemia. Lancet Oncol (2020) 21(10):e477–87. doi: 10.1016/S1470-2045(20)30342-9 33002443

[22] Swoboda R Labopin M Giebel S Angelucci E Arat M Aljurf M . Total body irradiation plus fludarabine versus thiotepa, busulfan plus fludarabine as a myeloablative conditioning for adults with acute lymphoblastic leukemia treated with haploidentical hematopoietic cell transplantation. a study by the acute leukemia working party of the EBMT. Bone Marrow Transplant (2022) 57(3):399–406. doi: 10.1038/s41409-021-01550-0 35031709

[23] Peters C Dalle JH Locatelli F Poetschger U Sedlacek P Buechner J . Total body irradiation or chemotherapy conditioning in childhood ALL: A multinational, randomized, noninferiority phase III study. J Clin Oncol (2021) 39(4):295–307. doi: 10.1200/JCO.20.02529 33332189 PMC8078415

[24] Marks DI Wang T Pérez WS Antin JH Copelan E Gale RP . The outcome of full-intensity and reduced-intensity conditioning matched sibling or unrelated donor transplantation in adults with Philadelphia chromosome-negative acute lymphoblastic leukemia in first and second complete remission. Blood (2010) 116(3):366–74. doi: 10.1182/blood-2010-01-264077 PMC2913452 20404137

[25] Ringdén O Labopin M Ehninger G Niederwieser D Olsson R Basara N . Reduced intensity conditioning compared with myeloablative conditioning using unrelated donor transplants in patients with acute myeloid leukemia. J Clin Oncol (2009) 27(27):4570–7. doi: 10.1200/JCO.2008.20.9692 19652066

[26] Bornhäuser M Kienast J Trenschel R Burchert A Hegenbart U Stadler M . Reduced-intensity conditioning versus standard conditioning before allogeneic haemopoietic cell transplantation in patients with acute myeloid leukaemia in first complete remission: A prospective, open-label randomised phase 3 trial. Lancet Oncol (2012) 13(10):1035–44. doi: 10.1016/S1470-2045(12)70349-2 22959335

[27] Scott BL Pasquini MC Logan BR Wu J Devine SM Porter DL . Myeloablative versus reduced-intensity hematopoietic cell transplantation for acute myeloid leukemia and myelodysplastic syndromes. J Clin Oncol (2017) 35(11):1154. doi: 10.1200/JCO.2016.70.7091 28380315 PMC5455603

[28] Nakasone H Fukuda T Kanda J Mori T Yano S Kobayashi T . Impact of conditioning intensity and TBI on acute GVHD after hematopoietic cell transplantation. Bone Marrow Transplant (2015) 50(4):559–65. doi: 10.1038/bmt.2014.293 25531281

[29] Yamamoto M Itou T Okikawa Y . Effectiveness of intensity-modulated radiation therapy (IMRT) for total body irradiation (TBI). Rinsho Hoshasen (2014) 59(7):988–95.

[30] Stein AS Al Malki MM Yang D Palmer J Tsai N-C Aldoss I . Total marrow and lymphoid irradiation (TMLI) at a dose of 2000cGy in combination with post-transplant cyclophosphamide (PTCy)-based graft versus host disease (GvHD) prophylaxis is safe and associated with favorable GvHD-Free/Relapse-Free survival at 1 year in patients with acute myeloid leukemia (AML). Blood (2020) 136:41–2. doi: 10.1182/blood-2020-141469

[31] Kanakry CG Tsai H-L Bolaños-Meade J Smith BD Gojo I Kanakry JA . Single-agent GVHD prophylaxis with posttransplantation cyclophosphamide after myeloablative, HLA-matched BMT for AML, ALL, and MDS. Blood J Am Soc Hematol (2014) 124(25):3817–27. doi: 10.1182/blood-2014-07-587477 PMC4263989 25316679

[32] Rühm W Azizova T Bouffler S Cullings HM Grosche B Little MP . Typical doses and dose rates in studies pertinent to radiation risk inference at low doses and low dose rates. J Radiat Res (2018) 59(suppl_2):ii1–ii10. doi: 10.1093/jrr/rrx093 29432579 PMC5941142

[33] Konishi T Ogawa H Najima Y Hashimoto S Wada A Adachi H . Safety of total body irradiation using intensity-modulated radiation therapy by helical tomotherapy in allogeneic hematopoietic stem cell transplantation: A prospective pilot study. J Radiat Res (2020) 61(6):969–76. doi: 10.1093/jrr/rraa078 PMC7674702 32888029

[34] Schultheiss TE Wong J Liu A Olivera G Somlo G . Image-guided total marrow and total lymphatic irradiation using helical tomotherapy. Int J Radiat OncolBiolPhys (2007) 67(4):1259–67. doi: 10.1016/j.ijrobp.2006.10.047 17336225

[35] Penagaricano J Chao M Van Rhee F Moros E Corry P Ratanatharathorn V . Clinical feasibility of TBI with helical tomotherapy. Bone Marrow Transplant (2011) 46(7):929–35. doi: 10.1038/bmt.2010.237 20935684

[36] Patel P Aydogan B Koshy M Mahmud D Oh A Saraf SL . Combination of linear accelerator-based intensity-modulated total marrow irradiation and myeloablative fludarabine/busulfan: A phase I study. Biol Blood Marrow Transplant (2014) 20(12):2034–41. doi: 10.1016/j.bbmt.2014.09.005 25234438

[37] Hill GR Crawford JM Cooke KR Brinson YS Pan L Ferrara JL . Total body irradiation and acute graft-versus-host disease: the role of gastrointestinal damage and inflammatory cytokines. Blood J Am Soc Hematol (1997) 90(8):3204–13. doi: 10.1182/blood.V90.8.3204 9376604

[38] Hill GR Ferrara JL . The primacy of the gastrointestinal tract as a target organ of acute graft-versus-host disease: Rationale for the use of cytokine shields in allogeneic bone marrow transplantation. Blood J Am Soc Hematol (2000) 95(9):2754–9. doi: 10.1182/blood.V95.9.2754.009k25_2754_2759 10779417

[39] Hui S Takahashi Y Holtan SG Azimi R Seelig D Yagi M . Early assessment of dosimetric and biological differences of total marrow irradiation versus total body irradiation in rodents. Radiother Oncol (2017) 124(3):468–74. doi: 10.1016/j.radonc.2017.07.018 PMC5624834 28778346

[40] Sullivan KM Goldmuntz EA Keyes-Elstein L Mcsweeney PA Pinckney A Welch B . Myeloablative autologous stem-cell transplantation for severe scleroderma. N Engl J Med (2018) 378(1):35–47. doi: 10.1056/NEJMoa1703327 29298160 PMC5846574

